# Adenocarcinomatous-predominant subtype associated with a better prognosis in adenosquamous lung carcinoma

**DOI:** 10.1186/s12885-020-06972-5

**Published:** 2020-06-05

**Authors:** Yangli Liu, Ying Zhu, Lihong Bai, Fengjia Chen, Jue Wang, Yubiao Guo

**Affiliations:** 1 Division of Pulmonary and Critical Care Medicine, the First Affiliated Hospital of Sun Yat-sen University, Guangzhou, 510080 Province Guangdong People’s Republic of China; 2 Department of Radiology, the First Affiliated Hospital of Sun Yat-sen University, Guangzhou, 510080 Province Guangdong People’s Republic of China; 3 Institution of Precision Medicine, The First Affiliated Hospital of Sun Yat-sen University, Guangzhou, 510080 Province Guangdong People’s Republic of China; 4 Department of Pathology, the First Affiliated Hospital of Sun Yat-sen University, Guangzhou, 510080 Province Guangdong People’s Republic of China

**Keywords:** Lung adenosquamous carcinoma, Surgery resection, Pathological subtypes, Prognosis

## Abstract

**Background:**

According to the proportion of glandular and squamous pathological components, adenosquamous carcinoma (ASC) could be divided into adenocarcinoma (AC) and squamous cell carcinoma (SCC) predominant subtypes. Due to its rarity, no study investigating the impact of different subtypes on the clinical features, radiologic findings and prognosis characteristics of ASC has been reported.

**Methods:**

Sixty eight patients who underwent surgical resection for lung adenosquamous carcinoma in our institute between January 2006 and March 2017 were retrospectively reviewed. Data regarding the clinical features, radiologic findings and prognosis characteristics were collected.

**Results:**

Thirty nine patients of the study cohort were with AC-predominant ASC and 29 with SCC-predominant ASC. There was no significant difference between the two subgroups in age, gender, smoking history, serum carcinoembryonic antigen (CEA) level and T,N classification. Air bronchogram was found more frequently in AC-predominant ASC than in SCC-predominant ASC (*P* = 0.046). Multivariate analysis identified pathological subtype (*P* = 0.022) and CT findings of peripheral location (*P* = 0.009) to be independent prognostic factors.

**Conclusions:**

AC-predominant ASC were more commonly presented with air bronchogram, and were with a better prognosis than SCC-predominant ASC.

## Background

Adenosquamous Carcinoma (ASC) is an uncommon type of lung cancer, accounting for 0.4–4% of all lung carcinomas [1, 2]. According to the 2015 WHO lung tumor classification criteria, it is defined as “a carcinoma showing components of both squamous cell carcinoma (SCC) and adenocarcinoma (AC), with each comprising to at least 10% of the tumor” [3]. However, ASC lung tumors were much more complex than simple mixes of AC and SCC components which were classified as intermediate between the AC and SCC when classifying the histological subtypes using genetic or molecular method [4]. ASC was reported to have more aggressive behavior and worse prognosis than “pure” AC and SCC with more frequent lymph-node metastasis at diagnosis [5].

Morphological features of AC and SCC were well described, AC being with acinar, lepidic, micropapillary, or papillary structures while SCC with identifiable keratinization, pearl formation, and/or intercellular bridges [3]. According to the proportion of glandular and squamous components, ASC could be divided into AC and SCC predominant subtypes. A few study reported that predominant subtype to be associated with prognosis of ASC [6, 7]. However, due to its rarity, no definitive clinical conclusion have been reached and to the best of our knowledge, there has been no report regarding radiologic findings of ASC with different predominant subtypes. Therefore, in this study we further explored the impact of different subtypes on the clinical features, radiologic findings and prognosis characteristics of ASC.

## Methods

### Patients

We reviewed the medical records database of patients diagnosed with ASC between January 2006 to March 2017 in our institution. Of 108 such patients, 33 patients without surgical resection of tumor were excluded, 7 patients with loss of follow-up were further excluded. 68 cases were included in this study and were retrospectively reviewed. According to the 2015 WHO Classification of Lung Tumors, ASC is defined as a carcinoma with both AC and SCC components ≥10% of the tumor [3]. Staging was based on the criteria of the 7th edition of the tumor, node, metastasis (p-TNM) classification for lung cancer [8]. This study was approved by the Ethical Committee of Human Experimentation in the First Affiliated Hospital of Sun Yat-sen University.

### Pathologic studies

The histopathological specimens were independently examined by two senior clinical pathologists. Immunohistochemical analysis was performed in all cases to identify the AC and SCC components. Patients with ASC were divided into two groups according to extent of the AC component. When the AC component was less than or equal to 50%, the ASC was considered SCC predominant, and when the AC component was more than 50%, the ASC was considered AC predominant.

### Follow up

Clinical manifestations, CT findings, treatment and prognostic data were collected. Follow-up information for all patients was obtained by telephone call. Overall survival (OS) was defined as the time interval between the day of surgery and the date of death from any cause or the last follow-up date.

### Statistical analysis

Statistical analysis was performed with SPSS 19.0 (SPSS, Chicago, IL). Student t test or Wilcoxon’s rank sum test were used for comparison between groups. Categorical variables were compared using contingency table analysis and χ2 tests. Cumulative survival was calculated using the Kaplan-Meier product method, the log rank-test was used to calculate differences. Univariate predictors were considered to be significant with a probability (*p*) value of < 0.05 and entered into a stepwise multivariable model assessed by the Cox proportional hazards model. A *P*-value < 0.05 was considered statistically significant.

## Results

### Patient characteristics

Patient clinical characteristics were reported in Table 1. Complete tumor resection with hilar mediastinal lymphadenectomy was accomplished in all patients. ASC was more prevalent among men (*n* = 46, 67.6%) than among women (*n* = 22, 32.4%) (*p* ≤ 0.01). The mean age of the patients at the time of diagnosis was 58.6 years. More (*n* = 38, 55.9%) patients were with history of smoking. All cases were classified as stages I to IIIA, according to the seventh edition of the TNM classification system [8]. Most of the tumors were in T1 ~ 2 (*n* = 54, 79.4%). Lymph node metastasis (N1 ~ 2) was found in 50 (73.5%) of the 68 tumors. Stage I ~ II, III tumors were found in 38(55.9%) and 30 (44.1%) patients, respectively. Most of the patients underwent lobectomy (*n* = 56, 82.4%), 2 patients underwent wedge resection and 10 patients (14.7%) underwent extended resection (including 6 bilobectomy and 4 pneumonectomy).

**Table 1:** Clinicopathologic features of patients with AC-predominant and SCC-predominant adenosquamous carcinoma

Variable	ASC Total (N,%)	AC-predominant (*n* = 39)	SCC-predominant (*n* = 29)	*p*-value
Age(y)	58.6 ± 11.5	59.9 ± 10.1	57.6 ± 12.6	0.096
Gender				0.746
Male	46 (67.6%)	27	19	
Female	22 (32.4%)	12	10	
Smoking history				0.276
Yes	38 (55.9%)	24	14	
No	30 (44.1%)	15	15	
CEA (ng/ml)	6.40 (3–20.36)	8.47 (3–28.73)	5.07 (2.43–14.4)	0.327
T-stage				0.986
T1 ~ T2	54 (79.4%)	31	23	
T3	14 (20.6%)	8	6	
N-stage				0.857
N0	18 (26.5%)	10	8	
N1 ~ 2	50 (73.5%)	29	21	
TNM-stage				0.919
I ~ II	38 (55.9%)	22	16	
IIIA	30 (44.1%)	17	13	
Surgical procedures				0.291
Wedge	2 (2.9%)	2	0	
Lobectomy	56 (82.4%)	30	26	
Extended resection	10 (14.7%)	7	3	

*AC* adenocarcinoma, *SCC* squamous cell carcinoma, *CEA* carcinoembryonic antigen

**Fig. 1:**
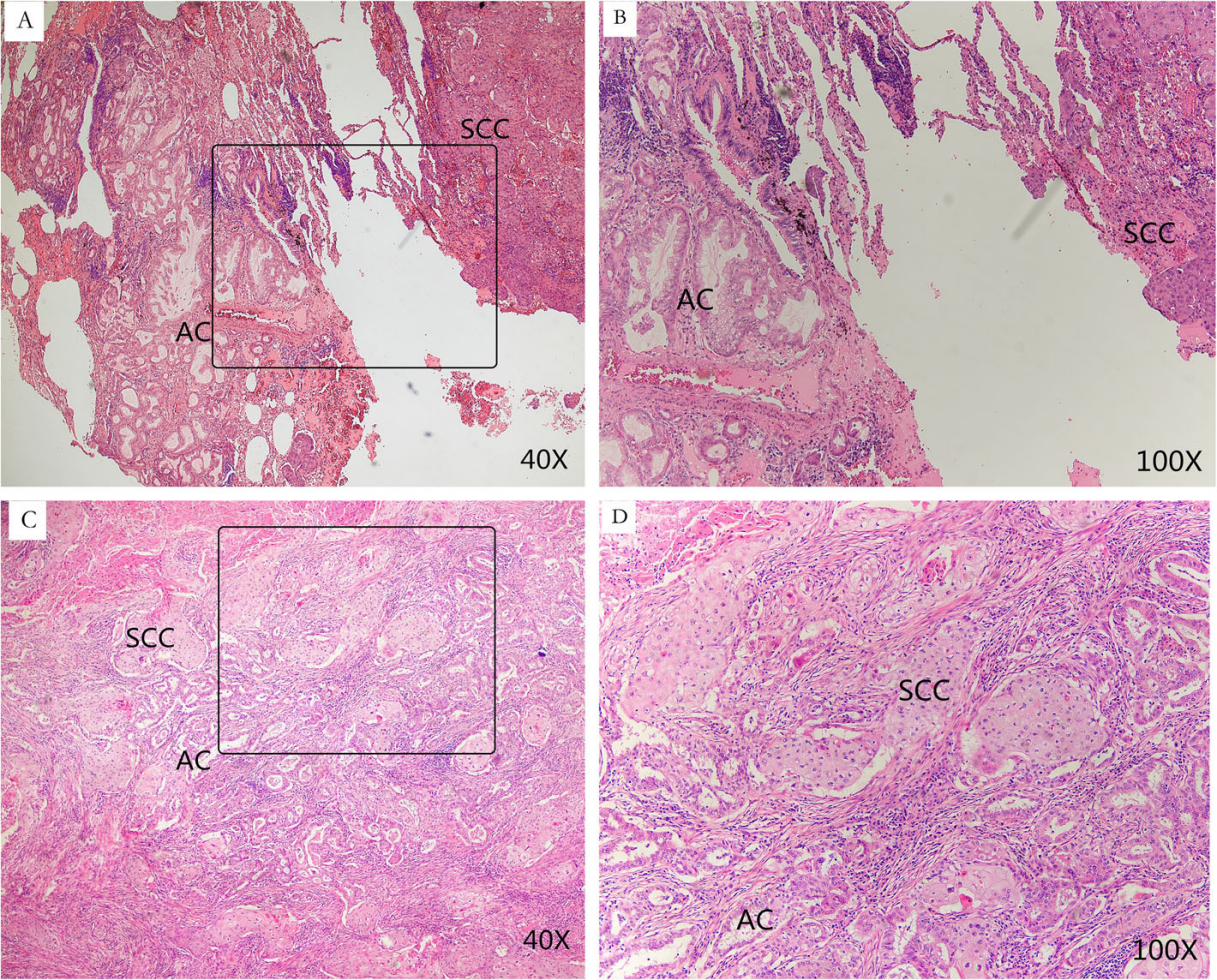
**a, b**- Representative histological images of AC-predominant ASC of the lung. **c, d**- Representative histological images of SCC-predominant ASC of the lung

Thirty nine patients of the study cohort were with AC-predominant ASC and 29 with SCC-predominant ASC (Fig. 1a-d). No statistically significant difference was found in age, gender, smoking history, serum carcinoembryonic antigen (CEA) level, surgical procedures and T,N classification between the two subgroups.

### CT findings of the tumor

Tumor diameter ranged from 12 to 70 mm [40.9 ± 11.8, mean ± standard deviation (SD)] and tended to be positioned in peripheral part of the affected lobe (*n* = 50, 73.5%). Thirty-three (48.5%) of the tumors were round or oval and 29 (42.6%) were with smooth contours. Margin characteristics were as follows: lobulation in 44 (64.7%), spiculation in 42 (61.8%), and pleural tag in 34 (50.0%). With regard to internal characteristics, air bronchogram was seen in 33 (48.5%), calcification in 18 (26.5%), cavitation in 24 (35.3%), and vessel covergence in 36 cases (52.9%).

Air bronchogram was found more frequently in AC-predominant ASC than in SCC-predominant ASC (*n* = 23, 58.9% vs. *n* = 10, 34.4%, *P* = 0.046). No statistical significance was noted in the maximum diameter, location, shape, contours, margin and other internal characteristics in both subtypes. The comparison of CT characteristics between AC-predominant and SCC-predominant ASC is summarized in Table 2.

**Table 2:** CT characteristics with AC-predominant and SCC-predominant adenosquamous carcinoma

Characteristic	ASC Total (N,%)	AC- predominant (*n* = 39)	SCC- predominant (*n* = 29)	*p*-value
Max diameter (mm)	40.9 ± 11.8	40.4 ± 11.3	41.5 ± 12.1	0.411
Location				0.857
Central	18 (26.5%)	10	8	
Peripheral	50 (73.5%)	29	21	
Shape				0.132
Round or oval	33 (48.5%)	22	11	
Irregular	35 (51.5%)	17	18	
Contours				0.754
Smooth	29 (42.6%)	16	13	
Irregular	39 (57.4%)	23	16	
Margin				
Lobulation	44 (64.7%)	28	16	0.156
Spiculation	42 (61.8%)	25	17	0.654
Pleural tag	34 (50.0%)	21	13	0.462
Internal characteristics				
Air bronchogram	33 (48.5%)	23	10	0.046
Calcification	18 (26.5%)	10	8	0.857
Cavitation	24 (35.3%)	14	10	0.904
Vessel covergence	36 (52.9%)	22	14	0.506

*AC* adenocarcinoma, *SCC* squamous cell carcinoma

### Survival analyses

The median postoperative follow-up was 27.5 months (range, 4–72 months). OS rates of all stage ASC cases were 53.5% at 3 years and 25.6% at 5 years. The 5-year OS rates based on T status were as follows: T1 24.6%, T2 19.8% and T3 7.1% (*P* = 0.115). OS rates for stage I, II, and IIIA ASC patients at 5 years were: 33.6, 5.0, 23.0%, respectively (*P* = 0.103).

The associations of various prognostic factors with postoperative survival using univariate analysis are presented in Table 3, which showed that structural components (*P* = 0.031), tumor location (*P* = 0.011) were significantly associated with OS (*P* < 0.05). Median overall survival time was 35 months vs. 24 months for AC-predominant ASC compared with SCC-predominant ASC and 5-year OS rates for AC-predominant ASC and SCC-predominant ASC were 25.6% vs. 5.6% (*P* = 0.031). For patients with centrally located ASC and peripherally located ASC, median overall survival time was 23 months vs. 35 months while 5-year OS rates were 5.6% vs. 23.8% (*P* = 0.011).

**Table 3:** Univariate analysis of different prognostic parameters in patients with AC-Predominant and SCC-Predominant Adenosquamous carcinoma

Variable	Median Overall Survival(m)	95%CI	*P* value
Age(y)			0.066
< 60	27	19.0–34.9	
≥ 60	38	23.2–52.8	
Gender			0.824
Male	31	19.8–42.2	
Female	30	16.4–43.6	
Smoking history			0.422
Yes	26	17.2–34.8	
No	35	22.7–47.3	
Structural components			0.031
AC-Predominant	35	25.6–44.4	
SCC-Predominant	24	12.4–35.6	
Tumor location			0.011
Peripheral	35	27.9–42.1	
Central	23	10.5–35.5	
T-stage			0.115
T1	38	24.2–51.8	
T2	34	28.8–39.2	
T3	23	15.6–30.3	
N-stage			0.238
N0	31	5.7–56.3	
N1	24	19.2–28.8	
N2	37	30.8–43.2	
TNM-stage			0.103
I	46	21.2–70.8	
II	26	19.8–32.2	
IIIA	32	20.9–43.0	

**Fig. 2:**
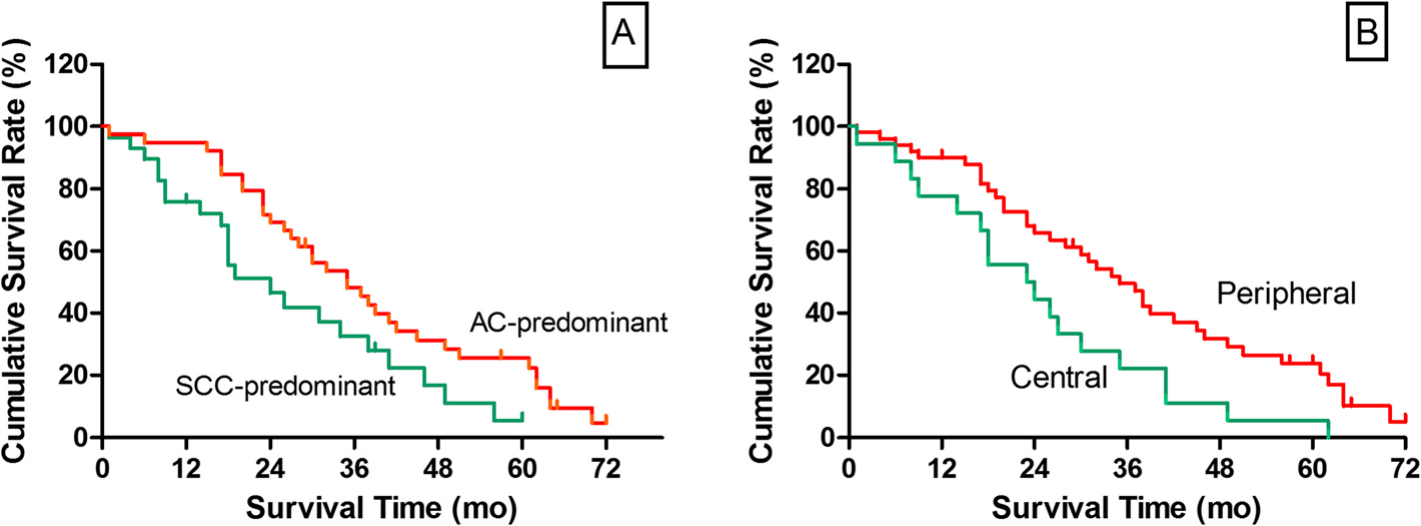
**a, b**- Overall survival (OS) analysis. **a** OS curves for patients with AC-predominant ASC and SCC-predominant ASC (*P* < 0.05). **b** OS curves for patients with peripherally located ASC and centrally located ASC. (*p* < 0.05)

Further multivariate analysis using the Cox’s proportional hazards model revealed the following to be independent prognostic factors: structural components (*P* = 0.022) and CT findings of peripheral location (*P* = 0.009). Patients with AC-predominant ASC exhibited a significantly better prognosis compared with patients with SCC-predominant ASC [HR 0.515 (0.29–0.91)]. In addition, patients with peripherally located ASC was associated with better survival outcomes [HR 0.462 (0.26–0.83)] (shown in Table 4). Figure 2 shows the Kaplan-Meier overall survival curves with the prognostic variables listed in Table 4.

**Table 4:** Independent Impacts of Variables on Patient: Overall Survival Estimated by Multivariate Analysis

Variable	*P* value	HR	95% CI
Structural components			
AC- vs. SCC-Predominant	0.022	0.515	0.29–0.91
Tumor location			
Peripheral vs. Central	0.009	0.462	0.26–0.83
Age(y)			
< 60 vs. ≥60	0.277

## Discussion

As far as we could establish, this is the first study to investigate the clinical, radiographic as well as prognostic characteristics of patients with the two different pathological subtypes of ASC. Our findings observed that: (1) ASC tended to have clinical characteristics of both adenocarcinoma and squamous cell carcinoma with poorer prognosis; (2) ASC could be divided into AC-predominant and SCC-predominant according to pathological subtypes, AC-predominant ASC were more commonly presented with air bronchogram and were with a better prognosis than SCC-predominant ASC; (3) Peripheral location of ASC served as an independent good prognostic factor.

Similar to previous reports, ASC tended to have the clinical characteristics of both adenocarcinoma and squamous cell carcinoma. On the one hand, imaging examination showed that most of the tumors were peripherally located, consistent with the characteristics of adenocarcinoma; On the other hand, most of the patients were males and smokers in their sixth decade of life, similar to squamous cell carcinoma. In this study, 73.5% (50/68) of the patients presented with peripherally located tumor, 67.6% (46/68) of the patients had a history of smoking, and the most common group was male smokers about 60 years old, which was consistent with previous studies [7, 9]. Our study also confirmed the invasive biological behavior of of ASC. The 3- and 5-year survival rates (53.5% at 3 years and 25.6% at 5 years) of all patients with stage I,II,IIIA were lower than those with AC or SCC. Even in Stage I and after complete surgical removal, the 5-year survival rate was only 33.6%. These were consistent with other reports [5, 10]: Maeda H [10] reported that the 5-year survival rates for all stage (I,II,IIIA) cases were 23.3% for ASC, 58.0% for AC (*p* < 0.0001), and 40.8% for SCC (p < 0.0001). The invasive biological behavior of ASC was also demonstrated by the high percentage of lymph node metastasis. We observed 50 (73.5%) of the 68 tumors had lymph node metastasis (N1 ~ 2).

To determine whether the predominant component affect clinical and radiographic characteristics, ASC cases were subdivided according to the predominance of AC or SCC, and differences between groups were evaluated. In our study, no significant differences were found between the two groups in age, gender, smoking history, CEA level and TNM stage. When it comes to CT findings in the subgroups, no literature has been reported. Here, we reported for the first time that AC-predominant ASC were more commonly presented with air bronchogram, which might be explained by previous report that the prevalence of air bronchograms on CT could predict the invasiveness of lung adenocarcinoma [11] since air bronchogram sign was formed when tumor cells lined the alveolar walls and alveolar septa, spreaded from one lobule to another through lymphatic, airways or direct infiltration, leaving the bronchi patent with mucosa intact [12]. This finding suggested that air bronchogram might be one of the predictive marker of AC-predominant ASC. When it is difficult for patients to take re-biopsy or when the biopsy specimens are insufficient for further pathological examination, the CT findings might be helpful in the differential diagnosis for pathological types, which may affect subsequent treatment.

Previous studies on the prognosis of ASC based on the proportion of adenomatous or squamous components have shown conflicting results. Takamori [13], Shimizu [14] and Filosso PL [15] suggested that the amount of adenocarcinoma component did not affect the survival rate, although the histological features of metastatic lymph nodes were to some extent affected by the histological type of the primary tumor. However, Gawrychowski [9], Mordant [16] and Zhao H [6] observed that patients who maintained a balance between the two ASC histological components had a better prognosis than those with one predominant component. These conflicting results may be due to the low incidence of ASC and the different pathological grouping criteria, for example some studies divided ASC to 3 groups with the cutoff of 40 and 60% components [6, 9], others divided ASC to 2 or 3 groups with the cutoff of 50% components [13, 15, 16], so definitive clinical conclusions can only be reached through more prospective multicenter studies. In our present study, multivariate analysis using the Cox’s proportional hazards model adjusted for age, gender, smoking status and tumor stage revealed that structural components (*P* = 0.022) was significantly correlated with prognosis. Patients with AC-predominant ASC had a better prognosis than with SCC-predominant ASC (median OS time 35 months vs. 24 months and 5-year OS rates 25.6% vs. 5.6%). Previous studies have shown that the squamous cell and adenocarcinomatous components might be derived from the same cells [17], and then monoclonal squamous cell carcinoma transformed to adenocarcinoma in ASC [18]. And we hypothesized that AC-predominant ASC might be in a later, better-differentiated phase in this transition and thus be with a better prognosis. However, recent data suggested that Asian pulmonary ASC might originate from adenocarcinoma and that squamous cell carcinoma components might be transformed from adenocarcinoma components [19]. Thus, further research is needed to explore this issue.

Few reports have described the relationship between CT findings and prognosis of ASC. Lee Y [20] evaluated 26 patients with ASC and found that the central ASC was larger than the peripheral ASC but except for tumor size, they found no significant difference in pathology, FDG PET, and survival data. Watanabe Y [7] analyzed 52 patients with ASC and reported that CT findings of inflammatory changes surrounding the tumor rather than tumor location (peripheral or central) were strong predictors of poor prognosis. Interestingly, they did find that centrally located ASC tended to have more inflammatory changes surrounding the tumor. In this study with the largest population focused on CT findings of ASC, we found that peripheral location of ASC served as an independent good prognostic factor (*P* = 0.009). Further research is needed to explore the differences between the central and peripheral ASC and why peripheral location predicts better survival.

There are several limitations in our study such as the limited number of patients with ASC, the retrospective design, different chemotherapy regiments, and unknown driver gene mutation status, which might influence the prognosis data [21, 22]. Therefore, further studies are warranted.

## Conclusion

In conclusion, our study showed that the two different pathological subtypes of ASC were with different radiologic findings and prognosis characteristics. AC-predominant ASC were more commonly presented with air bronchogram, and were with a better prognosis than SCC-predominant ASC. Studies with a much larger sample size and longer duration of follow-up are still necessary to confirm these results.

## Data Availability

The datasets used during the current study are available from the corresponding author on reasonable request.

## Abbreviations

AC: Adenocarcinoma
ASC: Adenosquamous carcinoma
CEA: Carcinoembryonic antigen
CT: Computed tomography
HR: Hazard ratios
LN: Lymph node
OS: Overall survival
SCC: Squamous cell carcinoma
TNM: Tumor, node, metastasis
